# Ulceration of the Trachea Produced by the Presence of the Canula after Tracheotomy

**Published:** 1859-07

**Authors:** 


*Ulceration of the Trachea produced by the Presence of the Canula after Tracheotomy.*

In the *Académie de Médicine*, at the sitting of April 5th, Dr. Henri Roger gave a *résumé* of a memoir on this subject, which he deposited in the *bureau* of the Academy. We find this *résumé* in the *Gazette Hebdomadaire*,

This article is based upon twenty-four observations; two of which he obtained from the Foundling Hospital, six borrowed from different authors especially from M. Barthez, and thirteen of his own cases. This memoir finishes with the following conclusions:

"Among the accidents which follow tracheotomy practiced for croup, there is one which is now pointed out, but which has not been studied—*ulceration of the trachea by the canula.*

"Tracheal ulcer is a lesion sufficiently frequent, especially in certain epidemics of croup, since we have been able, at the childrens' hospital, to collect thirteen cases in less than three months, out of a number of sixty-three young subjects tracheotomized in the first three months of 1859.

"Pathologically, in an anatomical point of view, we must distinguish:

"1. Erosion of the mucous membrane.

"2. Ulceration, properly so called.

"3. Complete perforation of the wind-pipe.

"The tracheal ulceration is almost always seated at the anterior wall of the tube, on a level with the inferior edge of the vertical portion of the canula, and is produced by the friction made by the edge, a little bent and cutting, so as to permit it to bear against and penetrate the anterior wall of the trachea, in the movements of respiration and deglutition.

"Twice in twenty-one cases, the ulcer was situated exclusively at the *posterior wall*; and four times it occupied simultaneously the anterior and posterior walls of the trachea."

M. Roger goes on to give the remaining conclusions derived from this memoir; of which we will give merely an abstract. The ulceration is almost constantly single, but there may be several in the case of a strong epidemic influence. Its form is generally oval. Complete perforation of the trachea sometimes takes place. (Four times in twenty-one cases.)

The coincident pathological alterations, in the order of frequency, are: ulceration or diphtheritis of the wound in the neck; double broncho-pneumonia, tracheitis or bronchitis; suppuration of the surrounding tissues; and spontaneous multiple ulcerations of the air passage. The first symptoms which would lead us to suspect this accident are, a bad state of the external wound, the false membranes, ulceration and gangrene which develop themselves; a discoloration of the canula, fetidity of the breath and matter discharged from the canula, sometimes bloody expectoration, and, in some infants, a pain in the anterior part of the neck, with dysphagia. Such is the group of symptoms which permits us to establish the diagnosis of tracheal ulceration.

The primitive cause is traumatic; the accessory causes are, a congestional phlegmonous state of the respiratory passages (tracheal ulcerations being as frequent results to tracheotomy for croup as they are rare after this operation in chronic affections of the larynx), a general bad condition produced most frequently by a diphtheritic diathesis, and the tender age of the patients, who, not being docile, cause, in their unreflecting movements, the canula to rub continually against the mucous membrane of the trachea. The particular nature of the epidemic of diphtherite, which we have observed, has given us in the first three months of 1859, three times as many cases of tracheal ulceration, as were observed during the entire year of 1858.

The prognosis of tracheal ulceration presents a certain degree of gravity, varying with the extent of the lesion; whether it be an erosion, an ulceration, or a perforation; but the gravity is never such as should deter a surgeon from performing tracheotomy in cases where death is imminent, and that operation is the great resort.

The treatment is essentially preventative, and consists in the employment of a canula of small size, slightly oblique as it descends, or by a flexible canula. (The flexible canula of M. Luër is one which has appeared to us thus far, to best fulfill the indications. Of four patients on whom it was employed, one was cured, and two others are in the way of cure.) "From the first, and after the first days which follow the operation of tracheotomy, one will endeavor to extract the canula momentarily, in order to relieve the trachea, at least for some instants, from that cause of injury, always being guided in respect to the duration of time during which the wind pipe is left without an instrument, by the manner in which respiration is accomplished."

